# GISTs with *NTRK* Gene Fusions: A Clinicopathological, Immunophenotypic, and Molecular Study

**DOI:** 10.3390/cancers15010105

**Published:** 2022-12-23

**Authors:** Zi Cao, Jiaxin Li, Lin Sun, Zanmei Xu, Yan Ke, Bing Shao, Yuhong Guo, Yan Sun

**Affiliations:** 1 Department of Pathology, Tianjin Medical University Cancer Institute and Hospital, National Clinical Research Center for Cancer, Key Laboratory of Cancer Prevention and Therapy, Tianjin’s Clinical Research Center for Cancer, Tianjin 300202, China; 2 Shanghai OrigiMed Co., Ltd., Shanghai 201112, China

**Keywords:** gastrointestinal stromal tumor, neurotrophic tyrosine receptor kinase, gene fusion, *ETV6–NTRK3*

## Abstract

**Simple Summary:**

Wild-type GISTs are generally not sensitive to tyrosine kinase inhibitors. Tropomyosin receptor kinase inhibitors have been approved to be effective in multiple cancers with neurotrophic tyrosine receptor kinase (*NTRK*) fusions. Although *NTRK* fusions are rare in wild-type GISTs, the unambiguous diagnosis can bring clinical benefits to the patients. The immunohistochemistry staining of Pan-TRK, next-generation sequencing or fluorescence in situ hybridization have been used to screen *NTRK* fusions in a few cases of wild-type GIST, and each technique has its advantages and drawbacks. This study aimed to identify *NTRK* fusions in wild-type GISTs with the above three methods and explore the clinicopathological and genetic features of GISTs with *NTRK* functions based on our patients and the literature. The findings from this study provide new evidence to establish a clinical protocol for screening GISTs with *NTRK* fusions and an overall view of the clinicopathological characteristics of GISTs with *NTRK* fusions.

**Abstract:**

The most common mutations in gastrointestinal stromal tumors (GISTs) are *KIT* or *PDGFRA* mutations. Recently, neurotrophic tyrosine receptor kinase (*NTRK*) fusions have been reported in *WT* GISTs, which increased interest in introducing tropomyosin receptor kinase (TRK) inhibitors as treatments for GISTs with *NTRK* fusions. Hence, we aimed to screen *NTRK* fusions in *WT* GISTs; we used fluorescence in situ hybridization (FISH), next-generation sequencing (NGS), and immunohistochemistry (IHC) to screen *NTRK* fusions in 46 *WT* GISTs and evaluate each method. We further reviewed *NTRK* fusion-positive GISTs from the literature and performed clinical and pathological analyses; two GISTs with an *ETV6*-*NTRK3* fusion (5%) were identified, while only one (50%) was positive for Pan-TRK expression. On the other hand, among the six GISTs with Pan-TRK-positive expression, only one (17%) harbored *NTRK* fusion. The literature review revealed the strong consistency between FISH and NGS and the limited value of Pan-TRK IHC in screening *NTRK* fusions in GISTs. In addition, the clinical and pathological analysis showed that GISTs with *NTRK* rearrangement occurred less frequently in the stomach, were more frequently larger in size, and the epithelioid type presented with a higher risk of recurrence. The *NTRK3* fusion has been more common than the *NTRK1* fusion in GISTs to date; our study identified two *ETV6*-*NTRK3* fusions in 46 *WT* GISTs. Compared with FISH and IHC, NGS is preferred for screening *WT* GISTs, including *NTRK* rearrangements. However, since GISTs with *NTRK* fusions are rare, further studies including more samples and mechanistic investigations should be conducted in the future.

## 1. Introduction

Gastrointestinal stromal tumors (GISTs) are the most common mesenchymal tumors of the gastrointestinal tract, with an annual incidence of 15 cases per million [1,2]. The majority of GISTs occur in the stomach (60%), followed by the small intestine (35%). GISTs are considered to originate from interstitial Cajal cells [3], with *KIT* or *PDGFRA* mutations detected in approximately 85% of cases [4,5]. GISTs without *KIT* or *PDGFRA* mutations are considered wild-type (*WT*) GISTs that are generally not sensitive to tyrosine kinase inhibitors (TKIs). Approximately 20–40% of *WT* GISTs are succinate dehydrogenase (*SDH*)-deficient GISTs, which are predominant in children and young patients, particularly in females, and are exclusively found in the stomach [6,7]. Among non-*SDH*-deficient *WT* GISTs, approximately 15% of cases carry mutations in *BRAF* or *RAS* [8,9,10]. The GISTs without mutations in *KIT*, *PDGFRA*, *SDH*, or *BRAF/RAS/NF1* (*RAS*-Pathway) were termed ‘quadruple *WT*’ GISTs [11] whose diverse molecular alterations and tumor biological behaviors remain unclear. Recently, several molecular alterations, including the *PIK3CA* mutation, neurotrophic tyrosine receptor kinase (*NTRK*) fusions (*ETV6-NTRK3* and *LMNA-NTRK1*), *FGFR1* gene fusions (*FGFR1-HOOK3* and *FGFR1-TACC*), *BRAF* gene fusions (*BRAF-AGAP3* and *BRAF-MKRN1*) and *ALK* gene fusion (*CDC42BPB-ALK*), have been reported in ‘quadruple *WT*’ GIST [12,13,14,15,16,17,18].

The *NTRK* family consists of *NTRK1*, *NTRK2*, and *NTRK3*, which encode tropomyosin receptor kinase (TRK) A, B, and C, respectively. Oncogenic TRK activation is mainly caused by the fusion of *NTRK* genes and is involved in the pathogenesis of many tumors, including infantile fibrosarcoma [19], mesoblastic nephroma [20], acute myeloid and chronic eosinophilic leukemia [21,22], secretory breast carcinoma [23], mammary analog secretory carcinoma of the salivary gland [24], radiation-induced thyroid carcinoma [25], myofibroblast tumors [26] and *WT* GISTs [12,13,14,15,27]. TRK inhibitors, such as larotrectinib and entrectinib, have been approved to treat multiple cancers with *NTRK* fusions [28,29,30,31]. Although *NTRK* fusions were detected in *WT* GISTs at a low frequency, patients with GIST presenting *NTRK* fusions had a good chance of responding to treatment with TRK inhibitors, particularly patients with recurrent tumors that are resistant to TKIs. Accordingly, *WT* GISTs with *NTRK* gene fusions must be identified. Fluorescence in situ hybridization (FISH) was identified as the gold standard for assessing gene fusions [32] and recommended by the European Society for Medical Oncology (ESMO) Translational Research and Precision Medicine Working Group when the 5′ partners of NTRK fusions were expected at high frequency [33]. However, *NTRK* fusions are rare in GISTs and involve several fusions that must be tested using *NTRK1*, *NTRK2*, and *NTRK3* probes [34]. With the application of next-generation sequencing (NGS), especially RNA sequencing, some *NTRK* fusions were identified in GISTs without the validation of FISH assessment [14,15]. In addition, immunohistochemical (IHC) staining of Pan-TRK was used to indicate *NTRK* fusions in some types of tumors; however, inconsistencies between Pan-TRK IHC staining and FISH results for *NTRK* rearrangements have been reported [13,35]. Therefore, the efficiency of the aforementioned methods in screening *NTRK* fusions in GISTs must be evaluated. In this study, we performed IHC staining for Pan-TRK in 39 non-*SDH*-deficient *WT* GISTs and NGS in 36 GISTs without *KIT*/*PDGFRA*/*SDH*/*BRAF*/*RAS* mutations and validated the results by performing a FISH assessment. Based on our results and the cases from the literature, we further explored the clinicopathological and genetic features of GISTs with *NTRK* rearrangements.

## 2. Materials and Methods

### 2.1. Patient Selection

We reviewed the medical records of all patients with GIST who underwent resection and genetic testing in our center between October 2014 and December 2019. The inclusion criteria were as follows: (a) primary GISTs, (b) endoscopic or surgical resection, (c) a diagnosis of GIST confirmed by pathologists, and (d) *WT* GISTs diagnosed based on Sanger sequencing of *KIT* and *PDGFRA* mutations. The exclusion criteria were as follows: (a) patients who were suspected to have GISTs by clinicians but not confirmed by pathologists, (b) patients who received neoadjuvant therapy before surgery, and (c) patients with recurrent or metastatic GISTs. According to these criteria, 46 patients with *WT* GISTs were included in this study.

### 2.2. Immunohistochemistry

IHC was performed on formalin-fixed, paraffin-embedded (FFPE) tissue sections with a thickness of 4 µm using an automated staining instrument (BENCHMARKXT, Roche, Tucson, AZ, USA). Primary antibodies against CD117 (YR145, maxim-BIO, Fuzhou, China), DOG-1 (OTI1C6, ZSGB-BIO, Beijing, China), CD34 (EP88, ZSGB-BIO, Beijing, China), SDHB (MAB-0736, maxim-BIO, Fuzhou China), BRAF V600E (H03529, Roche Diagnostics, Tucson, AZ, USA), and Pan-TRK (EPR17341, Roche Diagnostics, Monza, Italy) were used. The testing and assessment were performed according to the manufacturer’s instructions for every biomarker. For Pan-TRK, nuclear, cytoplasmic, or membranous staining in more than 5% of tumor cells was considered positive [36]. All IHC-stained sections were interpreted by two experienced pathologists.

### 2.3. Sanger Sequencing

DNA was extracted from FFPE sections using the QIAamp DNA FFPE Tissue Kit (Qiagen, Valencia, CA, USA). The degree of DNA degradation and RNA contamination were evaluated by the agarose gel electrophoresis. The purity and concentrations of DNA were accessed by measuring the A260/A280 ratio using NanoDrop2000 (Thermo Scientific, Waltham, MA, USA). DNA was quantified using Qubit2.0 (Thermo Scientific, Waltham, MA, USA) and then amplified with the following primers (Table S1). PCRs were performed using a HotStarTaq Plus Master Mix Kit (Qiagen, Valencia, CA, USA), and PCR products were purified using an Axyprep PCR Cleanup Kit (Axygen, Union City, CA, USA), labeled with a BigDye Terminator v3.1 Cycle Sequencing Kit (Thermo Scientific, Waltham, MA, USA) and sequenced with a 3500 Genetic Analyzer (Thermo Scientific, Waltham, MA, USA). The raw data were analyzed with Sequencing Analysis Software v5.4.

### 2.4. Next-Generation Target Sequencing (DNA and RNA)

DNA was extracted using QIAamp DNA FFPE Tissue Kit (Qiagen, CA, USA) and quantified using Qubit dsDNA HS Assay Kit (Life Technologies, Carlsbad, CA, USA). The gDNA library was captured using a customized 671 gene individually-synthesized 5′-biotinylated DNA 120 bp oligonucleotides probe panel with xGen Hybridization and quantified using Qubit dsDNA HS Assay Kit. RNA was extracted using a miRNeasy FFPE Kit (Qiagen, Hilden, Germany) and quantified using Qubit RNA HS Assay Kit (Thermo Fisher, Waltham, MA, USA). The cDNA library was captured using a customized 632 gene individually-synthesized 5′-biotinylated DNA 120 bp oligonucleotides probe panel with xGen Hybridization and Wash Kit (IDT, Coralville, IA, USA). The captured libraries were sequenced on Illumina NovaSeq 6000 with 2 × 150 bp paired-end reads, following the manufacturer instructions (Illumina, San Diego, CA, USA). A comprehensive genomic analysis of the sample was performed with a DNA + RNA cancer-related gene panel (YuanSu S, 671 DNA and 632 RNA gene panel, OrigiMed, Shanghai, China). DNA-seq reads were mapped to the hg19 reference sequence with BWA (version 0.7.12). PCR duplicates were removed by Pi-card (version 2.5.0), and recalibrated by the BaseRecalibrator tool from GATK (version 3.1.1). The in-house-developed algorithm was used for DNA fusion detection [37]. RNA-seq reads were using STAR (version 2.5.3) algorithm for mapping and STAR-fusion (version 0.8) for fusion detection [38]. Gene fusions were identified when total number of supportive reads spanning the fusion junction ≥5 [39].

### 2.5. Fluorescence In Situ Hybridization

FISH tests were performed on FFPE tissue sections with a thickness of 4 µm. Procedures, including denaturation at 73 °C for 5 min and hybridization at 37 °C for 16 h, were automated on a ThermoBrite hybridizer (Abbott, Chicago, IL, USA). DAPI (517,529, Abbott, Chicago, IL, USA) was used for counterstaining. Hybridization signals were analyzed under an Olympus BX53 (Olympus, Tokyo, Japan) fluorescence microscope. The probe signals were counted in at least 200 tumor cell nuclei per slide at 1000× magnification. The *ETV6-NTRK3* fusion probes (F.01258-01, LBP, Guangzhou, China) consisting of a spectrum green-labeled *NTRK3* (15q25) probe and a spectrum red-labeled *ETV6* probe (12q13.2) were used to identify the fusion of *ETV6* and *NTRK3*. The *ETV6-NTRK3* fusion was indicated by fused red and green signals in more than 10% of tumor cell nuclei [40]. *NTRK1* (220,101, HealthCare Biotechnology, Wuhan, China), *NTRK2* (220,501, HealthCare Biotechnology, Wuhan, China) and *NTRK3* (220,401, HealthCare Biotechnology, Wuhan, China) break-apart probes were used to detect the rearrangement of *NTRK1*, *NTRK2* and *NTRK3*, respectively. The *NTRK* rearrangements were interpreted by the presence of separated green and orange signals in more than 15% of tumor cell nuclei [27,41].

## 3. Results

### 3.1. Preliminary Testing of WT GISTs

**Figure 1:**
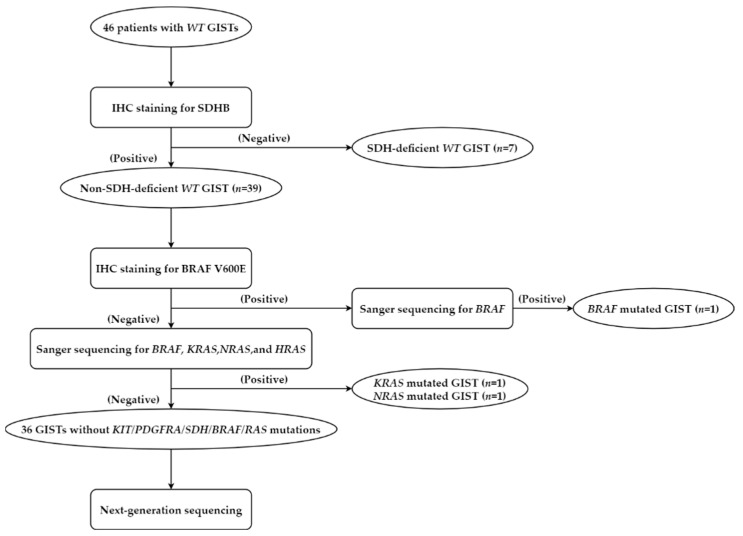
Flow diagram of preliminary testing of *WT* GISTs.

The 46 *WT* GISTs were divided into SDH-deficient (*n* = 7, Figure S1A) and non-SDH-deficient (*n* = 39, Figure S1B) groups based on the expression of SDHB. We performed IHC staining for BRAF V600E and Pan-TRK and Sanger sequencing for *BRAF*, *KRAS*, *NRAS*, and *HRAS* in the 39 non-SDH-deficient *WT* GISTs. One patient was positive for BRAF V600E (Figure S2A), followed by confirmation with Sanger sequencing for BRAF (c.1799 T > A, p.Val600Glu, Figure S2B). In addition, a *KRAS* exon 3 mutation (c.193A > G, p.Ser65Gly) and *NRAS* exon 2 mutation (c.35G > A, p.Gly12Asp) were detected in each patient (Figure S3A,B). Finally, we obtained 36 cases of GISTs without *KIT*/*PDGFRA*/*SDH*/*BRAF*/*RAS* mutations after IHC staining and Sanger sequencing, in which NGS was performed. No mutations of *KIT*, *PDGFRA*, *SDHA*, *SDHB*, *SDHC*, *SDHD*, *BRAF*, *KRAS*, *NRAS* or *HRAS* were detected by NGS in these 36 cases, which were consistent with the above results of Sanger sequencing and IHC. The Flow diagram of the preliminary testing of *WT* GISTs was presented in Figure 1.

### 3.2. NTRK Fusions Identified with NGS

**Figure 2:**
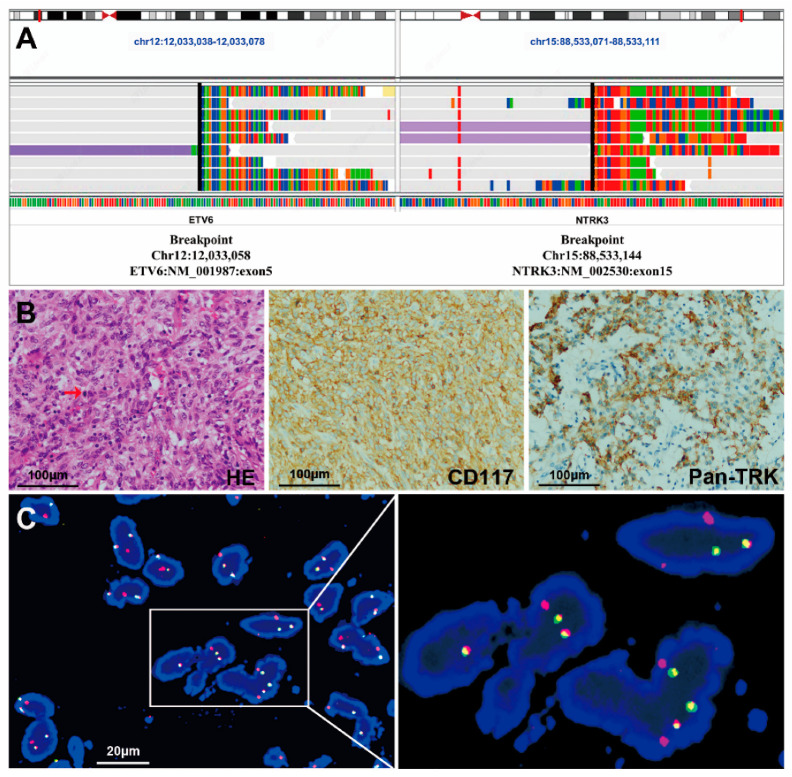
IGV, HE staining, immunohistochemical staining and FISH images of a mesenteric GIST with an *ETV6-NTRK3* fusion (case #1). (**A**) IGV images of NGS showed the *ETV6-NTRK3* fusion. (**B**) The tumor was composed of spindle-shaped or short spindle-shaped cells, and mitosis was observed (red arrow) (HE staining, 400×). Immunohistochemical staining showed strong positive expression of CD117 (400×) and Pan-TRK (400×). Each scale bar is 100 μm. (**C**) The FISH assay showed a pair of fused red and green signals using *ETV6-NTRK3* fusion probes in almost all tumor cells. Scale bar is 20 μm.

**Figure 3:**
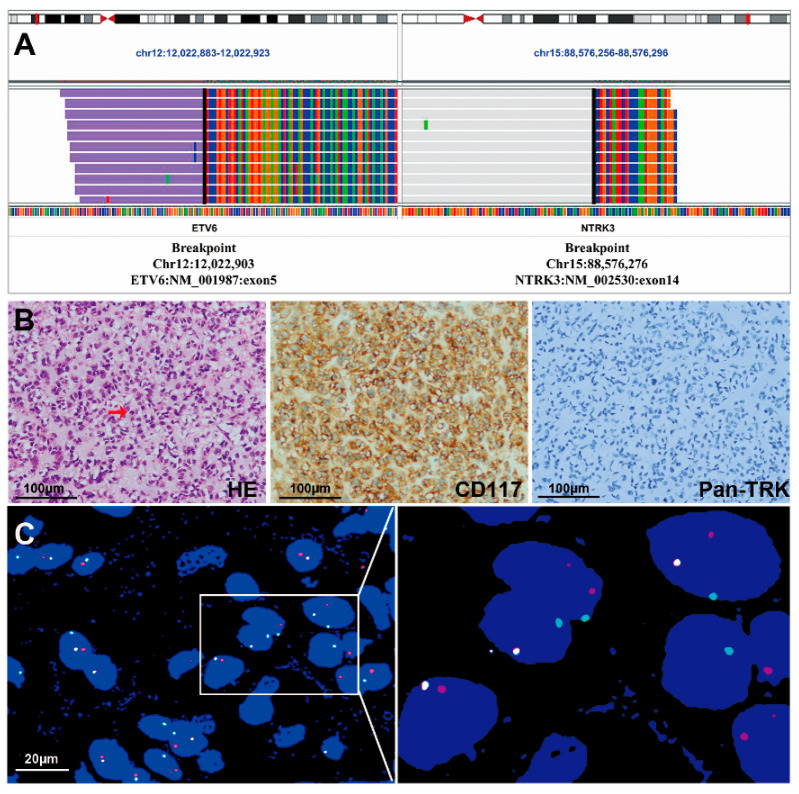
IGV, HE staining, immunohistochemical staining and FISH images of a gastric GIST with an *ETV6-NTRK3* fusion (case #2). (**A**) IGV images of NGS indicated an *ETV6-NTRK3* fusion. (**B**) HE staining showed that the GIST was composed of epithelioid cells, and the red arrow indicates tumor cell mitosis (HE staining, 400×). The tumor cells were positive for CD117 (400×) and negative for Pan-TRK (400×). Each scale bar is 100 μm. (**C**) The FISH assay showed a pair of fused red and green signals using *ETV6-NTRK3* fusion probes in almost all tumor cells. Scale bar is 20 μm.

*NTRK* fusions were detected with NGS in two samples from the 36 GISTs without *KIT/PDGFRA/SDH/BRAF/RAS* mutations. *ETV6-NTRK3* fusion was identified by DNA NGS (74 reads), showing that exon 1 to exon 5 of *ETV6* were fused with exon 15 to exon 19 of *NTRK3* in case #1 (Figure 2A). No gene rearrangement was detected using RNA-NGS in this case. The second case (case #2) was indicated to contain an *ETV6-NTRK3* fusion using RNA NGS (215 reads), showing that exon 1 to exon 5 of *ETV6* was fused with exon 14 to exon 19 of *NTRK3* (Figure 3A), but the gene fusion was not detected using DNA NGS.

### 3.3. IHC Staining of Pan-TRK

**Figure 4:**
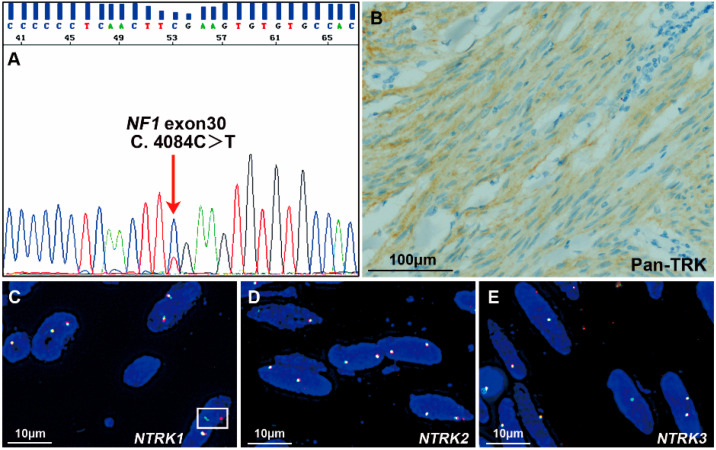
A *NF1*-related GIST with weak-moderate expression of Pan-TRK (case #3). (**A**) Sanger sequencing indicated a heterozygous point mutation in exon 30 of the *NF1* gene (c.4084C > T, p.Arg1362*). (**B**) IHC staining showed weak-moderate cytoplasmic expression of Pan-TRK (400×). Scale bar is 100 μm. (**C**–**E**) FISH images obtained using *NTRK1*, *NTRK2*, and *NTRK3* break-apart probes. *NTRK1* break-apart signals (in the white rectangle) were captured in only a few tumor cells (<15%), which could not be diagnosed as an *NTRK1* rearrangement. Neither *NTRK2* nor *NTRK3* rearrangements were detected in this tumor. Each scale bar is 10 μm.

Among the 39 non-SDH-deficient *WT* GISTs, Pan-TRK was negative in 33 tissues and positive in 6 samples (Table 1), including strong cytoplasmic expression in 3 tumors (cases #1, #4 and #5) and weak-moderate cytoplasmic expression in 3 tumors (cases #3, #6 and #7). Among the 3 tumors with strong Pan-TRK expression, an *ETV6-NTRK3* fusion was identified by NGS in only one case, which was a spindle-type GIST (case #1, Figure 2B). On the other hand, case #2 with an *ETV6-NTRK3* fusion identified by NGS was negative for Pan-TRK (Figure 3B). In addition, the 3 tumors with weak-moderate cytoplasmic Pan-TRK expression were all negative for *NTRK* rearrangement according to NGS. Among them, the *NF1* mutation was indicated by NGS and confirmed using Sanger sequencing (Figure 4A) in one tumor (case #3) showing weak-moderate expression of Pan-TRK (Figure 4B), and this patient was diagnosed with *NF1* syndrome based on the clinical characteristics.

### 3.4. FISH Assessments

For the two patients (cases #1 and #2) with *ETV6-NTRK3* fusions that were identified using NGS described above, we performed a FISH analysis using *ETV6-NTRK3* fusion probes. FISH assays confirmed the fusion of *ETV6* and *NTRK3* as a result of the (12p13.2;15q25) chromosome translocation in both cases (Figure 2C and Figure 3C).

**Figure 5:**
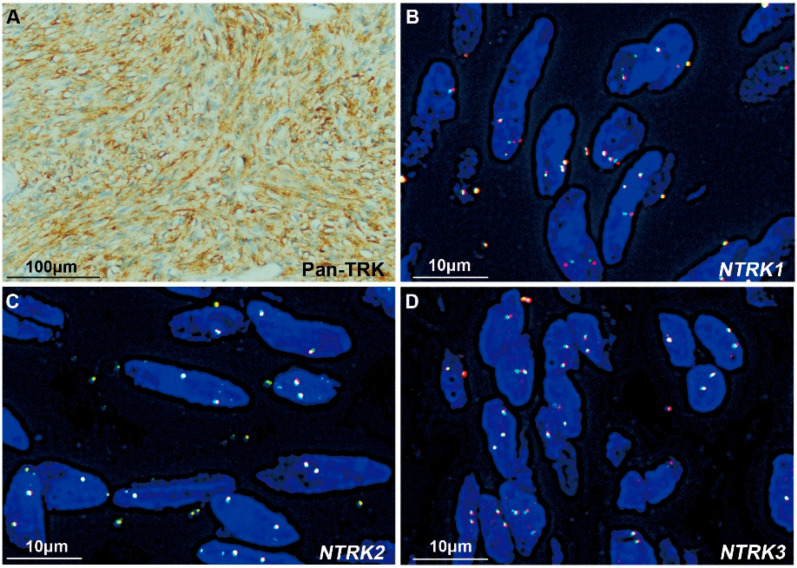
The tumor (case #4) with strong Pan-TRK expression but without *NTRK1*, *NTRK2* or *NTRK3* rearrangement. (**A**) IHC staining showed strong cytoplasmic expression of Pan-TRK (400×). Scale bar is 100 μm. (**B**–**D**) FISH images obtained using *NTRK1*, *NTRK2*, and *NTRK3* break-apart probes. The tumor was negative for *NTRK1*, *NTRK2* and *NTRK3* rearrangements. Each scale bar is 10 μm.

**Figure 6:**
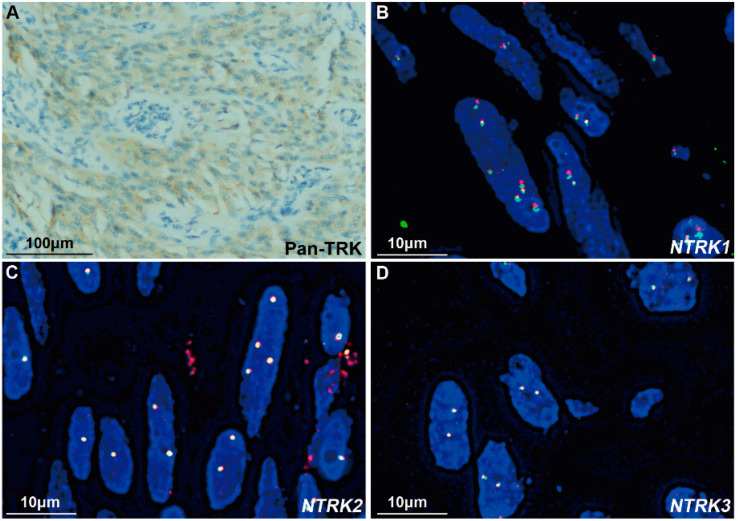
The tumor (case #6) with moderate expression of Pan-TRK and polyploidy involving *NTRK1*. (**A**) IHC staining showed moderate cytoplasmic expression of Pan-TRK (400×). Scale bar is 100 μm. (**B**–**D**) FISH images obtained using *NTRK1*, *NTRK2,* and *NTRK3* break-apart probes. Polyploidy, but not rearrangement, was observed in some tumor cells using the *NTRK1* break-apart probe. The tumor was negative for *NTRK2* and *NTRK3* rearrangements. Each scale bar is 10 μm.

We subsequently performed FISH assessments with *NTRK1*, *NTRK2*, and *NTRK3* break-apart probes in the 6 tumors with Pan-TRK positive expression. In case #1 with *ETV6-NTRK3* fusion and strong Pan-TRK expression, *NTRK3* was shown to be fragmented using the *NTRK3* break-apart probe, consistent with the results obtained using the *ETV6-NTRK3* fusion probe. Among the other two tumors with strong Pan-TRK expression, the *NTRK1*, *NTRK2* or *NTRK3* rearrangement was negative in one tumor (case #4, Figure 5), and no rearrangement was evaluated because of the poor DNA quality in the other sample (case #5). Among the 3 tumors with weak-moderate expression of Pan-TRK, case #3 was diagnosed as *NF1*-related GIST, as described above, in which *NTRK1* break-apart was captured in only a few tumor cells (<15%, Figure 4C) and could not be diagnosed as an *NTRK1* rearrangement. Moreover, this tumor was negative for *NTRK2* and *NTRK3* rearrangements (Figure 4D,E). Polyploidy was detected in most tumor cells using the *NTRK1* break-apart probe in case #6, but no rearrangement of *NTRK1*, *NTRK2*, or *NTRK3* was detected using the three break-apart probes (Figure 6). Another tumor (case #7) with weak-moderate expression of Pan-TRK was negative for *NTRK1*, *NTRK2* and *NTRK3* rearrangements (Figure S4). The FISH results were also shown in Table 1.

### 3.5. Comparisons among the Results of NGS, FISH, and IHC

Finally, we identified two GISTs (cases #1 and #2) with *NTRK* rearrangements among 39 non-SDH-deficient *WT* GISTs (5%). As shown in Table 1, the NGS and FISH results were absolutely consistent in screening GISTs with *NTRK* rearrangements. However, only one GIST (case #1) with a *NTRK* rearrangement (50%) was positive for Pan-TRK with IHC. On the other hand, among the six GISTs with Pan-TRK-positive expression, only one (case #1, 17%) contained an *NTRK* fusion, as determined using NGS or FISH.

### 3.6. The Clinicopathological Features of the Two GISTs with the ETV6-NTRK3 Fusion

In this study, case #1 with the *ETV6-NTRK3* fusion was a 52-year-old female patient who occasionally found a pelvic mass. The pelvic ultrasound scan showed an irregular mass of 13.3 × 13.8 × 6.6 cm in front of the anterior uterine wall with an unclear boundary. As the tumor had a rich blood supply and adhered extensively to the adjacent organs and vessels, cytoreductive surgery was performed to remove the mesenteric mass in November 2018 in our center. The gross appearance of the mass was a solid tumor with a largest diameter of 10.0 cm, gray-yellow-tan color, and tough texture. Microscopically, the tumor was composed of spindle or short-spindle cells (Figure 2B). Tumor cells surrounded thin- and thick-walled blood vessels in some areas, and hyalinization and myxoid matrix background were observed in some regions. The mitoses of tumor cells were obvious, with a mitotic rate of 8/5 mm^2^ and a Ki-67 index of 30%. Except for *ETV6-NTRK3* fusion, none of clinically significant mutations or fusions were found by NGS (Table S2). In addition, case #1 was indicated as microsatellite stable (MSS), and tumor mutation burden (TMB) was 4.4. The patient did not receive any adjuvant therapy based on the request of the patient and her family. The patient died 11 months after surgery.

Case #2 with *ETV6-NTRK3* fusion was a 56-year-old male patient who presented with intermittent epigastric pain in December 2017. An upper abdomen computed tomography scan revealed a mass of 16.2 × 9.5 × 8.0 cm in the lower part of the gastric body. The intraoperative exploration revealed a large mass with a size of 16.0 × 10.0 × 8.0 cm originating from the greater curvature of the gastric body and invading the mesocolon transversum, spleen, and pancreas. The surgeon performed a complete margin negative (R0) resection. Microscopically, hyalinized, and myxoid matrix backgrounds were observed in some tumor areas. Most of the tumor cells were epithelioid (Figure 3B). The mitotic index was 3/5 mm^2^ and the Ki-67 index was 8%. Although Pan-TRK staining was negative (Figure 3B), NGS and FISH detected an *ETV6-NTRK3* fusion (Figure 3A,C). Similar to case #1, except for *ETV6-NTRK3* fusion, no clinically significant mutations or fusions were found by NGS in case #2 (Table S2). In addition, case #2 was indicated as MSS, and TMB was 5.5. The patient received adjuvant treatment with 400 mg/qd imatinib and his disease did not progress during the follow-up period (58 months).

### 3.7. The Clinicopathological and Genetic Features of 12 GISTs with NTRK Fusions

We searched the related literature in PubMed to determine the clinicopathological and molecular characteristics of GISTs with *NTRK* fusions. We only found 10 GISTs with *NTRK* fusions from five papers thus far [12,13,14,15,27]. We summarized the clinicopathological parameters, immunohistochemical profile, and genetic features of the previously reported 10 GISTs (cases #8–#17) together with our two patients with *NTRK* fusions (cases #1 and #2) in Table 2. Among the 12 patients, the male: female ratio was 2:1. Except for one patient who was 20 years old, the other patients were all over 40 years old (50 ± 12 years old). The tumors were frequently found in the intestine (75%), including the rectum (4/12), duodenum (1/12), jejunum (1/12), colon (1/12), small bowel (1/12), and mesentery (1/12), followed by the stomach (16.7%) and unknown sites (8.3%). Except for the unknown tumor size in 3 patients, the maximum diameter of tumors was >10 cm in 3 patients (33.3%), 5.1–10.0 cm in 2 patients (22.2%), 2.1–5.0 cm in 3 patients (33.3%) and less than 2.0 cm in 1 patient (11.1%). Among the 4 patients with the information of localized or metastatic tumors, 3 patients (cases #1, #2, and #11) had localized tumors, and case #9 found metastasis at diagnosis. Probably due to metastasis at diagnosis, case #9 did not undergo surgery but received five lines of TKIs therapy. When the tumor progressed after five lines of TKIs, the patient was treated with a TRK inhibitor (larotrectinib) for 4 months and had an ongoing partial response (44%) according to the Response Evaluation Criteria in Solid Tumor (RECIST) version 1.114. Case #9 had been alive for 159 months at the end of the follow-up. Similar to case #9, case #10 did not undergo surgery but received four lines of TKI therapy. Case #10 had been alive for 12 months at the end of the follow-up. Unfortunately, case #12 died soon after the biopsy for diagnosis, so he did not undergo surgery or drug treatment. Except for the above 3 patients without surgery, 9 patients (cases #1, #2, #8, #11, #13~#17) underwent surgery. Among the 9 patients, 3 patients received adjuvant therapy with imatinib. Of the three patients with imatinib adjuvant therapy, 2 (cases #2 and #16) had no tumor progression at the end of follow-up, and another patient (case #15) died of tumor progression. Among the 6 patients who underwent surgery but not adjuvant therapy, 2 patients (cases #1 and #13) died of tumor progression, and 4 patients (cases #8, #11, #14, and #17) did not experience tumor progression and were alive at the end of follow-up. The treatment and follow-up of the 12 patients are also listed in Table 2.

Histologically, the epithelioid type was the most frequent (3/5, 60%), followed by the spindle type (1/5, 20%) and mixed spindle-epithelioid type (1/5, 20%), based on the information reported. Among the nine cases with known mitotic counts, the mitosis rate was >10/5 mm^2^ in four patients (44.4%), 5–10/5 mm^2^ in two patients (22.2%), and less than 5/5 mm^2^ in three patients (33.3%). According to the modified NIH risk classification [42], eight patients were classified as high risk (8/9, 88.9%), one patient was classified as extremely low risk (1/9, 11.1%) and the other three patients were unable to be classified without the information on tumor size and mitosis number. CD117 was positive in 9 patients (9/10, 90%), and DOG-1 was positive in all of the patients with known IHC results (10/10, 100%). All of the 12 GISTs with *NTRK* fusions were *WT* GIST without *KIT/PDGFRA* mutation. SDHB was positive in all of the patients with known results (5/5, 100%). IHC for Pan-TRK was performed in only 8 patients. Among them, Pan-TRK was positive in two patients (25%). NGS was performed in 7 patients, in which *ETV6-NTRK3* fusion was detected in 6 patients and *LMNA-NTRK1* fusion was identified in one patient. Among them, fusions were detected in 4 patients using DNA NGS and 3 patients using RNA NGS. FISH was performed in 9 patients. Among them, *ETV6-NTRK3* fusion probes were used in 2 patients (this study), both *NTRK3* break-apart probe and *ETV6* break-apart probe were used in one patient, *ETV6* break-apart probe was used in one patient, and *NTRK1* or *NTRK3* break-apart probe was used in 5 patients. Both NGS and FISH were conducted in four cases (cases #1, #2, #3 and #17) with consistent results. Finally, *NTRK3* fusion and *NTRK1* fusion were identified in 8 (66.7%) and 4 patients (33.3%), respectively.

## 4. Discussion

The *NTRK* genes encode TRK proteins that exert oncogenic effects on tumors, and the activation of most TRK proteins is caused by *NTRK* fusions [43]. *NTRK* fusions occur in a variety of cancers with different incidences [19,20,21,22,23,24,25,26,44]. TRK inhibitors, such as larotrectinib and entrectinib, showed encouraging antitumor efficacies in tumors with *NTRK* rearrangements and have been approved as treatments for multiple cancers harboring *NTRK* fusions [29,30,31,34,45,46,47]. Although the prevalence of *NTRK* fusions in GIST is extremely low and only a few patients were enrolled in the clinical trials, the antitumor efficacies of TRK inhibitors were observed [30,31]. In clinical trials (LOXO-TRK-14001, SCOUT, and NAVIGATE), 55 patients with tumors carrying *NTRK* rearrangements, including three GISTs, were enrolled to evaluate the efficacy of larotrectinib. All three patients with GIST experienced tumor shrinkage >30%, and one had a pathological complete response with sufficient tumor shrinkentrage >90% [30]. In trials (ALKA-372–001, STARTRK-1 and STARTRK-2) including 54 patients with different tumors carrying *NTRK* fusions (including one with *WT* GIST), entrectinib was reported to be beneficial in general, although the detailed information for the patient with GIST was not revealed [31]. In addition, one patient with GIST carrying an *ETV6-NTRK3* fusion received larotrectinib after five lines of TKIs (case #9 in Table 2) and achieved a partial response (44%) after 4 months of treatment [14]. Furthermore, larotrectinib has been recommended by the Belgian multidisciplinary expert panel as a first-line treatment for *WT* GIST with *NTRK* fusions [48]. These evidences suggest that *NTRK* fusions define a unique subgroup of GIST and TRK inhibitors have the potential to benefit GIST with *NTRK* fusions. Hence, it is clinically significant to screen *NTRK* fusions in *WT* GIST.

In this study, we identified *ETV6-NTRK3* fusion in two patients among 46 with *WT* GISTs using several methods. Our NGS and FISH results were absolutely consistent in screening *NTRK* rearrangements in GISTs. FISH has been recommended as the gold standard for detecting gene rearrangements, including break-apart probes and fusion probes. Break-apart probes are more broad-spectrum than fusion probes but do not supply information about partner genes involved in the fusions. In tumors with a high frequency of *NTRK* rearrangements, such as infantile fibrosarcoma, congenital mesoblastic nephroma, and secretory breast carcinoma, *NTRK1*, *NTRK2*, and *NTRK3* break-apart probes have been routinely used for the triple detection of FISH. In this study, we used *NTRK1*, *NTRK2*, and *NTRK3* break-apart probes, which were also used in other studies [27], to detect *NTRK* fusions in patients with Pan-TRK positive expression, but NGS did not show *NTRK* fusions. Considering the high cost of triple detection with three pairs of break-apart probes, this approach does not seem superior to screening *NTRK* fusions using FISH in GISTs, which are tumors with a low incidence of *NTRK* fusions. In this study, based on the detailed fusion information identified using NGS, we used *ETV6-NTRK3* fusion probes to verify the known fusions. As *ETV6* is the most frequent partner in *NTRK3* fusions, Castillon et al. sequentially used an *ETV6* break-apart probe and *NTRK3* break-apart probe to indicate the *ETV6-NTRK3* fusion in one *WT* GIST [13]. FISH appeared more suitable for verification than for screening gene fusions in tumors with a low incidence of the fusions.

Compared to FISH with specific probes, NGS may provide information on broad-spectrum molecular alterations in many genes, including mutations, amplification/deletion, gene fusions, and SNPs. Therefore, NGS has been recommended in *WT* GISTs and patients with GIST exhibiting acquired resistance [49,50,51,52]. Genomic profiling can be performed through DNA-based NGS in which DNA is stable, but it is impossible to design probes covering all introns since exons account for only a small proportion of the total gene; therefore, missed inspection is unavoidable for DNA-based NGS. This limitation is a probable cause of the lack of DNA-based NGS data for case #2 in this study. Compared with DNA-based NGS, RNA-based NGS completely covers all exons, which may decrease the probability of missed detection and can supply direct evidence of fusion proteins based on mRNA levels. However, RNA is unstable and easily degrades in FFPE samples, which decreases the efficiency of RNA-based NGS. We detected the *ETV6-NTRK3* fusion using DNA-based NGS but did not detect the fusion using RNA-based NGS in Patient One in the present study, which may result from the poor quality of RNA. Therefore, DNA + RNA NGS may reduce the false negative rate. In previous studies, some GISTs with the *ETV6-NTRK3* fusion were identified using RNA sequencing [12,27] and others were identified by performing genomic profiling of DNA [14]. NGS requires strict quality control and was not available in some primary hospitals. The doctors in those hospitals are suggested to refer the patients with non-SDH-deficient *WT* GIST to expert centers, so that the patients could gain appropriate treatment based on the comprehensive landscape of targeting alterations from NGS.

Compared with FISH and NGS, IHC is a cheap and fast testing method and is available in many medical centers. IHC staining for Pan-TRK was used to indicate *NTRK* fusions in several cancers [35,36,53,54,55]. However, both the sensitivity (50%) and specificity (16.7%) of Pan-TRK staining were low in our study. Moreover, in the 8 GISTs with *NTRK* rearrangement for which Pan-TRK results were available across several studies, only 2 tumors (25%) were positive for Pan-TRK. Therefore, the validity of Pan-TRK IHC staining in screening *NTRK* rearrangements was dubious in GISTs. In this study, polyploidy of *NTRK1* was detected in one tumor, and *NTRK1* break-apart signals were captured in a few tumor cells in another sample, which might be one explanation for the weak-to-intermediate expression of Pan-TRK. However, we could not explain the possible mechanism of the other two cases with strong or weak-to-intermediate expression of Pan-TRK but without any *NTRK* rearrangement, amplification, or mutation based on the current results of FISH and NGS. We also have no insights into the drug response to TRK inhibitors in patients with GIST presenting Pan-TRK-positive expression but not *NTRK* rearrangements. The relationship between Pan-TRK expression and *NTRK* alterations should be further studied, and more clinical trials are needed. However, considering the practicability, IHC staining for Pan-TRK is a cheap way to screen patients in a targeted population of non-SDH-deficient *WT* GIST if NGS is not available.

In this study, we also explored the clinicopathological and genetic features of GISTs with *NTRK* fusions based on our patients and the literature. Compared with the classic GISTs frequently observed in the stomach, more GISTs with *NTRK* fusions occurred in the intestine, especially in the rectum. GISTs with *NTRK* fusions tended to present as the epithelioid type, while approximately 70% of classic GISTs were the spindle type [56]. In addition, according to the current data, most GISTs with *NTRK* rearrangements had a high risk of recurrence. In addition, *NTRK3* fusions were more frequent than *NTRK1* fusions in GISTs based on the present data.

However, there were some limitations in our study. In the first place, we couldn’t provide further treatment information about the TRK inhibitors in our patients with *ETV6-NTRK* fusion, since one patient died before she had a chance to use TRK inhibitors and the other have no evidence to use TRK inhibitors according to the current status of no tumor recurrence. Secondly, although we described the patient’s survival status in detail in this study, we could not perform a survival analysis based on such small sample size. Furthermore, we used a pooled cohort to analyze the screening efficiency of IHC, NGS and FISH as well as the clinicopathological characteristics of the GISTs with *NTRK* fusions, but the information from different studies is inhomogeneous. The conclusions should be validated in multicenter studies with large sample size.

## 5. Conclusions

It is clinically significant to screen *NTRK* fusions in *WT* GIST. Among the techniques used for screening *NTRK* fusion, NGS and FISH presented strong consistency, while IHC staining for Pan-TRK had limited sensitivity and specificity. However, IHC is a cheap way to screen patients in a targeted population of non-SDH-deficient *WT* GIST if NGS is not available. On the other hand, NGS can allow a comprehensive landscape of targeting alterations and is recommended to be used in *WT* GISTs and the GISTs exhibiting acquired resistance. It is suggested to refer the patients with *WT* GIST to expert centers at the earliest practicable stage in their disease course for precise diagnosis and treatment. Based on the study in small sample size, GISTs with *NTRK* rearrangement less frequently occurred in the stomach, were more frequently large in size and presented with epithelioid type, and had a higher risk of recurrence. *NTRK3* fusion was more frequent than *NTRK1* fusion in GISTs thus far. However, since the GISTs with *NTRK* fusions are rare, further studies including more cases and mechanism investigations should be conducted in future.

## Figures and Tables

**Table 1:** FISH assessments in the cases with positive expression of Pan-TRK or *NTRK* fusion from NGS.

Case	IHC for Pan-TRK		NGS	FISH			
	Intensity	Positive Proportion	*NTRK* Fusions	*ETV6-NTRK3*	*NTRK3*	*NTRK2*	*NTRK1*
#1	Strong	50%	*ETV6-NTRK3*	Positive	Positive	Negative	Negative
#2	Negative	0	*ETV6-NTRK3*	Positive	NP	Negative	Negative
#3	Weak-moderate	70%	No ^a^	NP	Negative	Negative	Negative
#4	Strong	80%	No	NP	Negative	Negative	Negative
#5	Strong	50%	No	NP	NA ^b^	NA ^b^	NA ^b^
#6	Weak-moderate	30%	No	NP	Negative	Negative	Negative
#7	Weak-moderate	30%	No	NP	Negative	Negative	Negative

Abbreviations: NA: not available; ^a^: the *NF1* mutation was indicated by NGS and confirmed using Sanger sequencing; ^b^: no rearrangement was evaluated because of the poor DNA quality in the other sample, NP: not performed.

**Table 2:** Gastrointestinal Stromal Tumors with *NTRK* Gene Fusions Reported in the Literature.

Case	Age (Years), Sex	Location/ Localized or Metastatic	Size (cm)	Morphology	MI (/5 mm²)	Immunohistochemistry				*KIT/* *PDGFRA* Mutation	*NTRK* Fusion			Surgery	Drug Treatment	Progression/PFS (Months)	Status/OS (Months)	Reference
						CD117	DOG-1	SDHB	PTRK		Type	NGS	FISH					
#1	52, F	Mesentery/ Localized	10	S	8	P	P	P	P	No	*ETV6-NTRK3*	*DNA*-based	*ETV6-NTRK3*	Yes	No	Yes/11	Dead/11	this study
#2	56, M	Stomach/ Localized	16	E	3	P	P	P	N	No	*ETV6-NTRK3*	*RNA*-based	*ETV6-NTRK3*	Yes	*Imatinib*	No/58	Alive/58	this study
#8	44, M	Rectum/NA	5	E	34	P	P	P	NA	No	*ETV6-NTRK3*	*RNA*-based	*ETV6*	Yes	No	No/44	Alive/44	[12]
#9	55, M	Small bowel/Metastatic	NA	NA	NA	NA	NA	P	NA	No	*ETV6-NTRK3*	*DNA*-based	NA	No	*Imatinib*	Yes/NA ^a^	Alive/159	[14]
															*Sunitinib*			
															*Sorafenib*			
															*Nilotinib*			
															*Regorafenib Larotrectinib*			
#10	54, M	Colon/NA	NA	NA	NA	NA	NA	P	NA	No	*ETV6-NTRK3*	*DNA*-based	NA	No	*Imatinib Sunitinib* *Sorafenib* *Linsitinib*	Yes/NA ^b^	Alive/12	[14]
#11	20, M	Rectum/ Localized	7	S,E	10	N	P	NA	NA	No	*LMNA-NTRK1*	*DNA*-based	NA	Yes	No	No/7	Alive/7	[15]
#12	59, M	NA/NA	NA	E	NA	P	P	NA	N	No	*ETV6-NTRK3*	No	*NTRK3* and *ETV6*, respectively	No	No	No/0	Dead/0	[13]
#13	44, F	Rectum/NA	2.8	NA	17	P	NA	NA	Weakly positive in only one of the following five patients	No	*NTRK1*	No	*NTRK1*	Yes	No	Yes/108	Dead/132	[27]
#14	45, M	Duodenum/NA	1.7	NA	1	P	NA	NA		No	*NTRK3*	No	*NTRK3*	Yes	No	No/72	Alive/72	[27]

#15	65, F	Stomach/NA	17	NA	70	P	NA	NA		No	*NTRK1*	No	*NTRK1*	Yes	*Imatinib*	Yes/26	Dead/96	[27]
#16	61, F	Jejunum/NA	3.9	NA	12	P	NA	NA		No	*NTRK1*	No	*NTRK1*	Yes	*Imatinib*	No/48	Alive/48	[27]
#17	43, M	Rectum/NA	11	NA	0	P	NA	NA		No	*ETV6-NTRK3*	*RNA*-based	*NTRK3*	Yes	No	No/36	Alive/36	[27]

Abbreviations: M: male; F: female; S: spindled; MI: mitotic index; E: epithelioid; P: positive; N: negative; NA: not available; PFS: progression-free survival; OS: overall survival; ^a^ The patient experienced tumor progression during treatment with five lines of TKI therapy (155 months) and then achieved an ongoing partial response (44%) after larotrectinib therapy for 4 months. ^b^ The patient experienced tumor progression during treatment with four lines of TKI therapy (12 months).

## Data Availability

The datasets used and analyzed during the current study are available from the corresponding author on reasonable request.

## Supplementary Materials

The following supporting information can be downloaded at: https://www.mdpi.com/article/10.3390/cancers15010105/s1, Table S1:The primer sequences for exon amplification of target genes.; Table S2:The genetic alterations of the two GISTs with *ETV6-NTRK3* fusion through NGS; Figure S1: The expression of SDHB protein in wild-type GISTs; Figure S2: Pathological images and genetic testing results of a *BRAF*-mutant GIST; Figure S3: Pathological images and genetic testing results of two *RAS*-mutant GISTs; Figure S4: The tumor (case #7) with weak-moderate expression of Pan-TRK but without *NTRK1*, *NTRK2* or *NTRK3* rearrangement.
