# Supplementary material for: GISTs with NTRK Gene Fusions: A Clinicopathological, Immunophenotypic, and Molecular Study
Source: Cancers (Basel). 2022 Dec 23;15(1):105. doi: 10.3390/cancers15010105 (PMC9817796; doi:10.3390/cancers15010105)
Supplement: Supplementary file 1 [file cancers-15-00105-s001.zip › cancers-2037302-supplementary.pdf]

**Table S1.** The primer sequences for exon amplification of target genes.

| Gene-Exon | Forward Primer | Reverse Primer |
|---|---|---|
| BRAF-exon 15 | 5′-GGCCAAAAATTTAATCAGTGGA-3′ | 5′-TCATAATGCTTGCTCTGATAGGA-3′ |
| KRAS-exon 2 | 5′-AAAAGGTACTGGTGGAGTATTTGAT-3′ | 5′-AGCTGTATCGTCAAGGCACT-3′ |
| KRAS-exon 3 | 5′-AGGTGCACTGTAATAATCCAGAC-3′ | 5′-TGCATGGCATTAGCAAAGACTC-3′ |
| KRAS-exon 4 | 5′-AAGTTGTGGACAGGTTTTGAAAGA-3′ | 5′-AGAAGCAATGCCTCTCAAG-3′ |
| NRAS-exon 2 | 5′-GGCTCGCCAATTAACCCTGA-3′ | 5′-TCCGACAAGTGAGAGACAGGA-3′ |
| NRAS-exon 3 | 5′-GGTGAAACCTGTTTGTTGGACA-3′ | 5′-TCAGAACACAAAGATCATCCTTTCA-3′ |
| HRAS-exon 2 | 5′-GAGACCCTGTAGGAGGACCC-3′ | 5′-CTATCCTGGCTGTGTCCTGG-3′ |
| HRAS-exon 3 | 5′-GGATTCCTACCGGAAGCAGG-3′ | 5′-TCACGGGGTTCACCTGTACT-3′ |

Table S2: The genetic alterations of the two GISTs with *ETV6-NTRK3* fusion through NGS

| Case _ID | Gene_ Symbol | Transcript_ Ref | Alternation_ Type | Chromo some | Position_ Start | Position_ End | Coding_DNA _Change | AA_ Change |
|---|---|---|---|---|---|---|---|---|
| Case #1 | ALOX12B | NM_001139 | Substitution | chr17 | 7979002 | 7979002 | c.1565C>T | p.P522L |
| Case #1 | CIC | NM_015125 | Substitution | chr19 | 42791465 | 42791465 | c.453-7G>A | - |
| Case #1 | FGFR4 | NM_213647 | Substitution | chr5 | 176518037 | 176518037 | c.535A>G | p.T179A |
| Case #1 | FLT1 | NM_002019 | Substitution | chr13 | 29012441 | 29012441 | c.430G>A | p.E144K |
| Case #1 | JAK3 | NM_000215 | Substitution | chr19 | 17945482 | 17945482 | c.2248A>G | p.T750A |
| Case #1 | LRIG1 | NM_015541 | Substitution | chr3 | 66433702 | 66433702 | c.2195C>T | p.P732L |
| Case #1 | MAP3K1 | NM_005921 | Splice site | chr5 | 56183350 | 56183350 | c.4257+3G>A | - |
| Case #1 | MECOM | NM_001105077 | Substitution | chr3 | 168830581 | 168830581 | c.2202C>A | p.H734Q |
| Case #1 | MEF2B | NM_001145785 | Substitution | chr19 | 19256794 | 19256794 | c.919C>A | p.R307S |
| Case #1 | MST1R | NM_002447 | Substitution | chr3 | 49934990 | 49934990 | c.2009T>G | p.V670G |
| Case #1 | MUC16 | NM_024690 | Substitution | chr19 | 9020092 | 9020092 | c.37403G>A | p.S12468N |
| Case #1 | MUC16 | NM_024690 | Substitution | chr19 | 9076903 | 9076903 | c.10543C>T | p.H3515Y |
| Case #1 | MUC16 | NM_024690 | Substitution | chr19 | 9091783 | 9091783 | c.32C>G | p.S11C |
| Case #1 | OBSCN | NM_052843 | Substitution | chr1 | 228399525 | 228399525 | c.41C>G | p.T14S |
| Case #1 | PALB2 | NM_024675 | Substitution | chr16 | 23632742 | 23632742 | c.3054G>C | p.E1018D |
| Case #1 | PARP4 | NM_006437 | Substitution | chr13 | 25000682 | 25000682 | c.4901A>T | p.Q1634L |
| Case #1 | PRKCI | NM_002740 | Splice site | chr3 | 169953142 | 169953142 | c.223+3G>A | - |
| Case #1 | PTK2 | NM_001199649 | Substitution | chr8 | 141829049 | 141829049 | c.719T>C | p.I240T |
| Case #1 | RAD50 | NM_005732 | Substitution | chr5 | 131911578 | 131911578 | c.323A>G | p.K108R |
| Case #1 | RPTOR | NM_020761 | Substitution | chr17 | 78867500 | 78867500 | c.2243-7C>T | - |
| Case #1 | TERT | NM_198253 | Substitution | chr5 | 1272347 | 1272347 | c.2335C>T | p.H779Y |
| Case #1 | WEE1 | NM_003390 | Substitution | chr11 | 9606879 | 9606879 | c.1363A>C | p.N455H |
| Case #1 | XPO5 | NM_020750 | Substitution | chr6 | 43519108 | 43519108 | c.1655T>C | p.V552A |
| Case #2 | ARID1B | NM_020732 | Substitution | chr6 | 157521963 | 157521963 | c.4235C>T | p.S1412L |
| Case #2 | ARID1B | NM_020732 | Short indel | chr6 | 157100041 | 157100055 | c.993_1007del | p.G333_G337del |
| Case #2 | BCL2L11 | NM_138621 | Substitution | chr2 | 111881678 | 111881678 | c.356C>T | p.P119L |
| Case #2 | CCND1 | NM_053056 | Short indel | chr11 | 69465988 | 69465990 | c.839_841del | p.E280del |
| Case #2 | CHD2 | NM_001271 | Truncation | chr15 | 93498727 | 93498727 | c.1795del | p.L599Sfs*2 |
| Case #2 | FAT3 | NM_001008781 | Substitution | chr11 | 92087587 | 92087587 | c.2309C>T | p.T770M |
| Case #2 | FBXO31 | NM_024735 | Substitution | chr16 | 87367736 | 87367736 | c.1153G>A | p.G385R |
| Case #2 | FNDC3B | NM_022763 | Substitution | chr3 | 171969261 | 171969261 | c.720T>A | p.S240R |
| Case #2 | HCK | NM_001172133 | Substitution | chr20 | 30674516 | 30674516 | c.858C>A | p.N286K |
| Case #2 | IL7R | NM_002185 | Splice site | chr5 | 35871316 | 35871316 | c.537+1G>A | - |
| Case #2 | LRP1 | NM_002332 | Substitution | chr12 | 57561313 | 57561313 | c.2995+6A>T | - |
| Case #2 | MAGI2 | NM_012301 | Substitution | chr7 | 77649090 | 77649090 | c.3910G>A | p.G1304S |
| Case #2 | MLH1 | NM_000249 | Substitution | chr3 | 37067240 | 37067240 | c.1151T>A | p.V384D |
| Case #2 | MTOR | NM_004958 | Substitution | chr1 | 11307768 | 11307768 | c.1139G>C | p.C380S |
| Case #2 | NCOR1 | NM_006311 | Substitution | chr17 | 15995380 | 15995380 | c.2821-8T>C | - |
| Case #2 | NTRK1 | NM_002529 | Substitution | chr1 | 156851285 | 156851285 | c.2242C>T | p.R748W |
| Case #2 | PDCD1 | NM_005018 | Substitution | chr2 | 242794932 | 242794932 | c.277T>G | p.C93G |

| | | | | | | | | |
|---|---|---|---|---|---|---|---|---|
| Case #2 | SPTA1 | NM_003126 | Substitution | chr1 | 158613160 | 158613160 | c.4394A>T | p.H1465L |
| Case #2 | TERT | NM_198253 | Substitution | chr5 | 1279396 | 1279396 | c.2130+10G>A | - |
| Case #2 | ZNF750 | NM_024702 | Substitution | chr17 | 80789809 | 80789809 | c.522C>A | p.D174E |

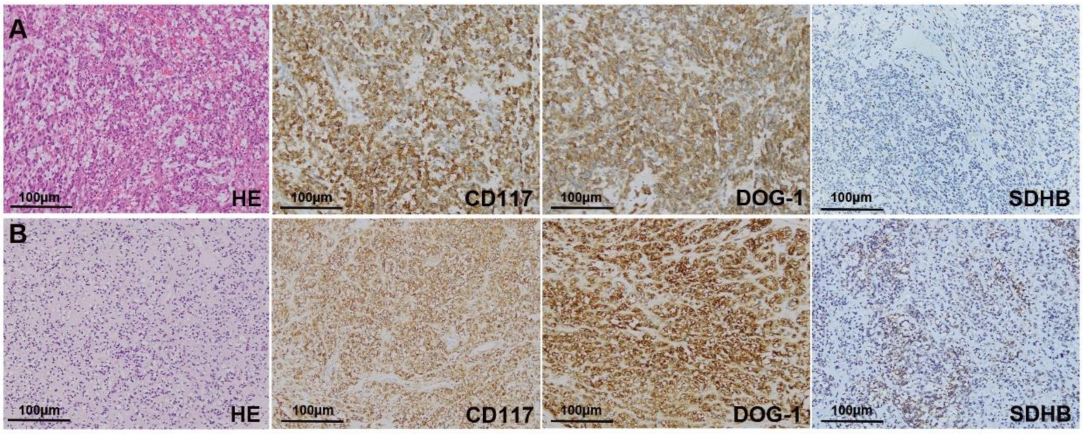


**Figure S1.** The expression of SDHB protein in wild-type GISTs. a A case of SDH-deficient GIST in gastric body. HE staining showed that tumor cells were epithelioid cell type (200×); IHC staining of CD117 (200×) and DOG-1 (200×) were positive. SDHB was negative in the tumor cells but positive in non-tumor cells (such as vascular endothelial cells, stromal cells and inflammatory cells) as internal control (200×). b A case of non-SDH-deficient GIST in gastric antrum, HE staining showed that tumor cells were epithelioid cell type (200×); IHC staining of CD117 (200×), DOG-1 (200×) and SDHB (200×) were positive in tumor cells. Each scale bar is 100μm.

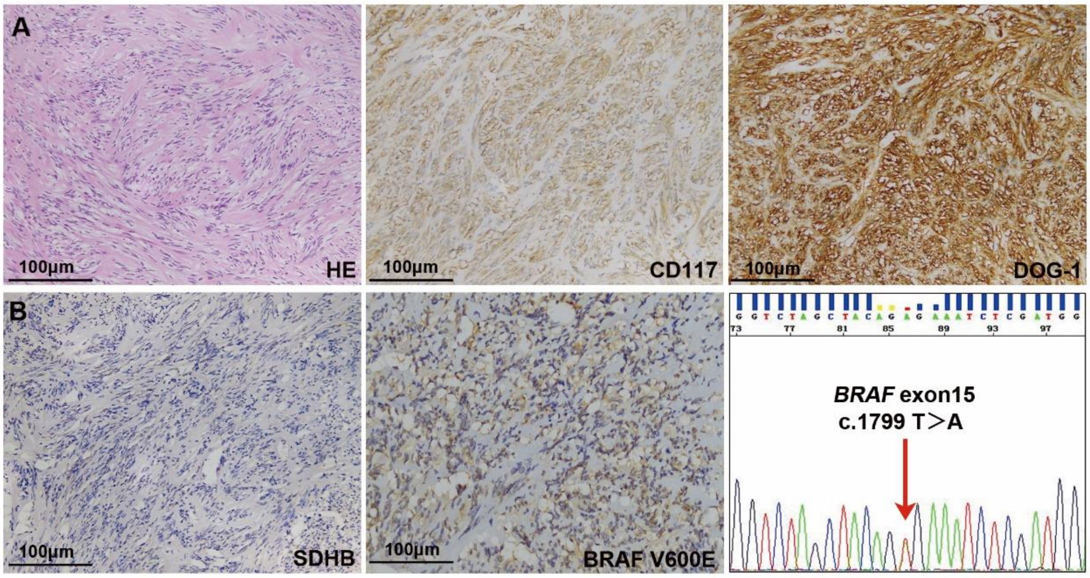


**Figure S2.** Pathological images and genetic testing results of a *BRAF*-mutant GIST. HE staining showed tumor cells were spindle cell type (200×); IHC staining showed that CD117 (200×), DOG-1 (200×), SDHB (200×) and BRAF V600E (400×) were all positive. The results of Sanger sequencing demonstrated a heterozygous point mutation in exon 15 of *BRAF* gene (c.1799 T>A, p. Val600Glu). Each scale bar is 100μm.

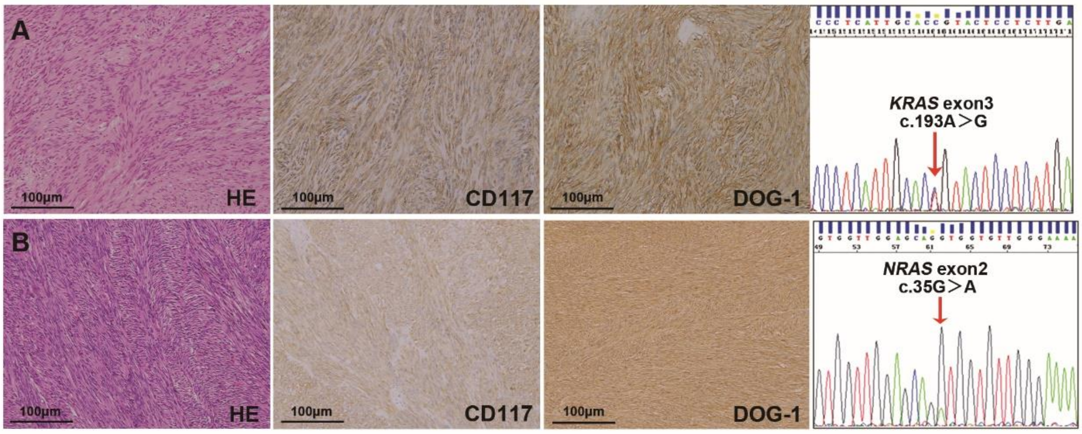


**Figure S3.** Pathological images and genetic testing results of two *RAS*-mutant GISTs. a A *KRAS*-mutant GIST in the junction between gastric body and antrum. HE image (200×) showed that the tumor cells were spindle cell type. IHC staining showed that CD117 (200×) and DOG-1 (200×) were positive. The results of Sanger sequencing showed a missense mutation in the exon 3 of *KRAS* gene (c.193A>G, p. Ser65Gly). b A *NRAS*-mutant GIST in gastric fundus and body. HE staining (200×) showed the tumor cells were spindle cell type. IHC staining showed that CD117 (200×) and DOG-1 (200×) were positive. The genetic testing result indicated a mutation in exon 2 of *NRAS* gene (c.35G>A, p. Gly12Asp). Each scale bar is 100μm.

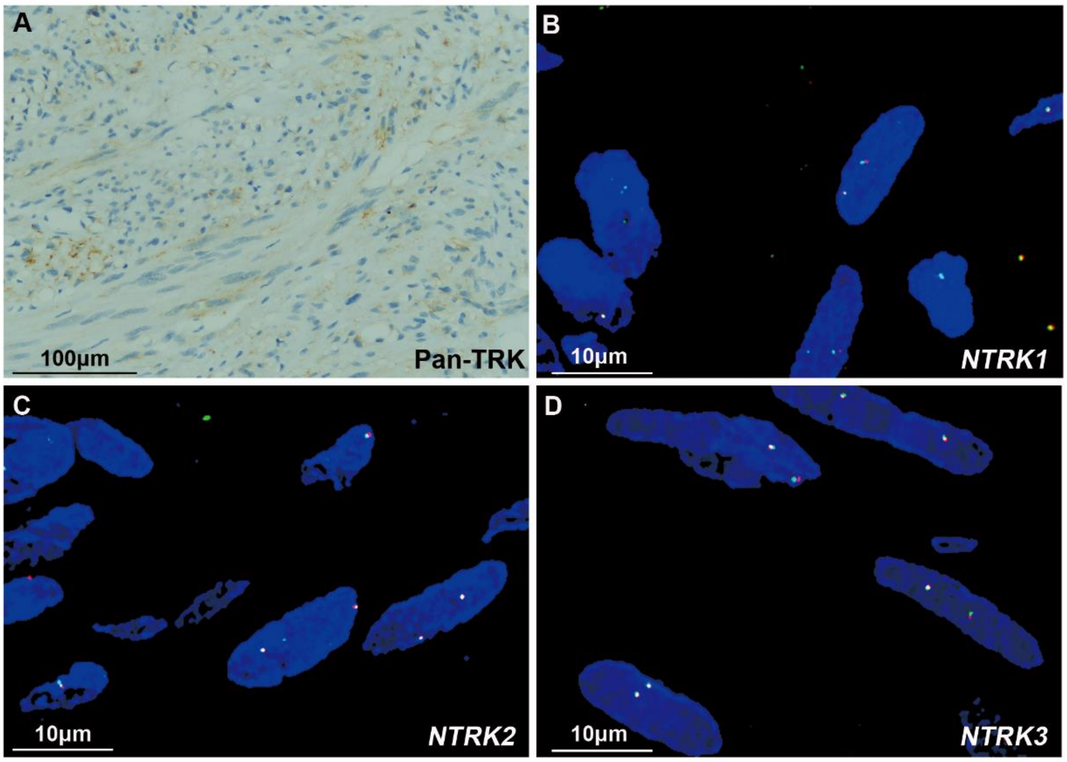


**Figure S4.** The tumor (case #7) with weak-moderate expression of Pan-TRK but without *NTRK1*, *NTRK2* or *NTRK3* rearrangement. a IHC staining showed weak-moderate cytoplasmic expression of Pan-TRK (400×). b-d None of *NTRK* rearrangements were detected by *NTRK1*/*2*/*3* break-apart probes. Each scale bar is 10μm.
